# Supplementary material for: Effects of Trehalose-6-Phosphate Synthase on the Reproduction and Development of Nilaparvata lugens and Its Molecular Mechanism
Source: Insects. 2025 Nov 24;16(12):1195. doi: 10.3390/insects16121195 (PMC12733863; doi:10.3390/insects16121195)
Supplement: Supplementary file 1 [file insects-16-01195-s001.zip › Text S1-Details for transcriptomics and metabolites.pdf]

Details for transcriptomics

(1) For total reads, raw reads ranged from 43.82 to 45.57 M, and clean reads (filtered by SOAPnuke) ranged from 41.98 to 43.59 M.

(2) For mapped reads, the genome mapping rate was 79.23–80.66% (HISAT2) with a unique mapping rate of 45.79–47.14%; the gene set mapping rate was 75.18–77.26% (Bowtie2) with a unique mapping rate of 61.75–66.09%.

(3) For coverage, the majority of transcripts had 90–100% transcript coverage, and reads showed a uniform distribution (with slight 3' bias).

(4) For quality score, base quality was ≥ Q20 (error rate ≤ 1%); the Pearson correlation coefficient for biological replicates was ≥ 0.8; sequencing saturation was achieved, with gene detection plateaued at 100×10^4 reads.

Key quality metrics for metabolites are as follows:

(1) A total of 1,768 compounds were detected in positive ion mode (pos), and 609 compounds were detected in negative ion mode (neg).

(2) Qualified compounds (with CV ≤ 30% in QC samples) included 1,574 in positive ion mode (pos), accounting for 89.03% of the total detected compounds, and 511 in negative ion mode (neg), accounting for 83.91% of the total detected compounds.

(3) Identification confidence was categorized into four levels—from Level 1 (highest, matching precursor ion, MS2 spectrum, and retention time) to Level 4 (only precursor ion match)—via the BMDB, mzCloud, and KEGG databases.
